# A Real-Time Imaging Algorithm Based on Sub-Aperture CS-Dechirp for GF3-SAR Data

**DOI:** 10.3390/s18082562

**Published:** 2018-08-05

**Authors:** Guang-Cai Sun, Yanbin Liu, Mengdao Xing, Shiyu Wang, Liang Guo, Jun Yang

**Affiliations:** 1 National Laboratory of Radar Signal Processing, Xidian University, Xi’an 710071, China; lyb_rsp@163.com (Y.L.); xmd@xidian.edu.cn (M.X.); 2 School of Physics and Optoelectronic Engineering, Xidian University, Xi’an 710071, China; yshi@mail.xidian.edu.cn (S.W.); lguo@mail.xidian.edu.cn (L.G.); 3 College of Geomatics, Xi’an University of Science and Technology, Xi’an 710054, China; yangjun_kx@163.com

**Keywords:** GF3-SAR, real-time, CS-dechirp, coherently combination, pipeline structure

## Abstract

Conventional synthetic aperture radar (SAR) imaging algorithms usually require a period of time to process data that is longer than the time it takes to record one synthetic aperture or that corresponding to an adequate azimuth resolution. That is to say, the real-time processing system is idle during the long data recording time and the utilization of computational resources is low. To deal with this problem, a real-time imaging algorithm based on sub-aperture chirp scaling dechirp (CS-dechirp) is proposed in this paper. With CS-dechirp, the sub-aperture data could be processed to form an image with relatively low resolution. Subsequently, a few low-resolution images are generated as longer azimuth data are recorded. At the stage of full-resolution image generation, a coherent combination method for the low-resolution complex-value images is developed. As the low-resolution complex-value images are coherently combined one by one, the resolution is gradually improved and the full-resolution image is finally obtained. The results of a simulation and real data from the GF3-SAR validate the effectiveness of the proposed algorithm.

## 1. Introduction

Spaceborne synthetic aperture radar (SAR) can perform two-dimensional high-resolution imaging of ground targets at a long distance and in all-weather and all-day conditions [1,2,3], which makes it a key method for real-time information acquisition. SAR has been widely used in battlefield reconnaissance, target identification, resource exploration, disaster detection, and many other important areas [4,5]. Real-time imaging processing is a key technology of spaceborne SAR in earth observation. To achieve real-time imaging, the processing system is required to produce the final image at the same time as data recording ends or with a relatively short delay [6]. Therefore, how to improve the real-time processing capability of spaceborne SAR is a key issue.

In ideal conditions, some classic algorithms such as the range Doppler algorithm (RDA) [7], chirp scaling algorithm (CSA) [8], range migration algorithm (RMA or omega-k algorithm) [9], and polar format algorithm (PFA) [10] can obtain well-focused images, which are usually used in SAR real-time processing. These algorithms usually process data in a time period longer than that required for one synthetic aperture or that corresponding to an adequate azimuth resolution [11,12]. That is to say, the real-time processing system is idle during the long data recording times and the utilization of computational resources is low. This problem is particularly obvious in spaceborne SAR systems due to their long radar range, high azimuth resolution, and thus long synthetic aperture length [13]. In this case, if the imaging processing starts after recording the azimuth data of a full-aperture time, the required data storage and computational load increases substantially [14,15], which undoubtedly increases the requirements for the hardware processing system of a spaceborne SAR and hinders real-time imaging processing.

To deal with this problem, a new sub-aperture approach for real-time SAR processing has been studied by Alberto Moreira [16]. This approach has shown good real-time performance and imaging results. The signal is divided into sub-apertures that are small enough, so the range cell migration in one sub-aperture datum can be ignored. However, the range cell migration is considered in sub-aperture signal stitching. The interpolation of samples is used for range cell migration correction in this approach. This correction is limited to migration within one sub-aperture, which is less than half of the range resolution. In addition, the overlap of the sub-apertures must be greater than 21% for sufficient attenuation of the grating lobes or paired echoes, which increases the computational load. As shown in the simulation, the grating lobes appear in the imaging results. In our opinion, this phenomenon occurs due to the overlapping sub-aperture data. On this basis, this paper proposes a real-time imaging algorithm based on sub-aperture chirp scaling dechirp (CS-dechirp), which can perform imaging processing while the data are recorded and thus without waiting for a full-aperture time. In the proposed method, the low-resolution complex-value image is formed by using sub-aperture data, which can be much shorter than a synthetic aperture length. Subsequently, a few low-resolution images are generated as the longer azimuth data is recorded. At the stage of full-resolution image generation, a coherent combination method for the low-resolution complex-value images is developed. As the low-resolution complex-value images are coherently combined one by one, the resolution is gradually improved and the full-resolution image is finally obtained. Compared with the study by Alberto Moreira [16], the method proposed in this paper does not require sub-aperture data to overlap, and the grating lobes did not appear in the simulation results. The range cell migration can be adequately corrected by the sub-aperture CSA without limitation. Since the processing system produces the image at the same time as recording the data, this pipeline structure is very suitable for the data stream in SAR real-time processing. 

This paper is organized as follows. In Section 2, the sub-aperture signal model for spaceborne SAR is established. In Section 3, the flow chart and equation derivation of the sub-aperture CS-dechirp imaging algorithm are detailed. In Section 4, the point targets simulation and the imaging results of the real data from the GF3-SAR are used to validate the effectiveness of the proposed algorithm. In Section 5, the content of this paper is summarized and analyzed.

## 2. Sub-Aperture Signal Model

**Figure 1:**
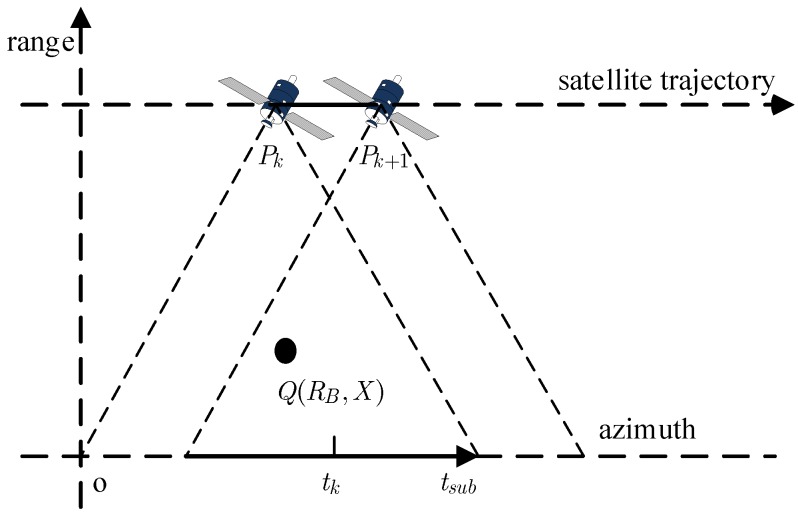
Geometry of the spaceborne synthetic aperture radar (SAR).

The geometry of the spaceborne SAR is depicted in Figure 1. The satellite moves along the trajectory from $P_{k}$ to $P_{k+1}$ for the k-th sub-aperture data recording, where $t_{k}$ is the center of the k-th sub-aperture data and $t_{sub}$ is the slow time in the azimuth of the sub-aperture data. According to Figure 1, the instantaneous slant range between the point target $Q(R_{B}^{},X)$ located in the scene and the ideal moving satellite can be written as:

(1) $$R(t_{sub};R_{B}^{})=\sqrt{R_{B}^{2}+{\left(vt_{k}+vt_{sub}-X\right)}^{2}}$$

where v is the equivalent speed of the spaceborne SAR, $R_{B}^{}$ is the coordinate in the range, and X is the coordinate in the azimuth of the point target $Q(R_{B}^{},X)$. 

Supposing that the transmitted signal is a linear frequency modulation (LFM) signal $s_{t}(t)=a_{r}(t)\exp(j\pi \gamma t^{2})$, where γ is the chirp rate of the LFM signal, $t_{}$ is the fast time in the range, and $a_{r}(t)$ is the window function of the LFM signal, which is a rectangular window [17], then the received sub-aperture signal of the point target $Q(R_{B}^{},X)$ can be written in the $t_{}$–$t_{sub}$ domain as:

(2) $$\begin{array}{cc} s(t,t_{sub};R_{B}^{}) & =a_{r}(t-\frac{2R(t_{sub};R_{B}^{})}{c})a_{a}(t_{k}+t_{sub})\\ & \times \exp[j\pi \gamma (t-\frac{2R(t_{sub};R_{B}^{})}{c}){}^{2}]\exp[-j\frac{4\pi }{\lambda }R(t_{sub};R_{B}^{})] \end{array}$$

where $a_{a}(\cdot )$ is the beam window function in the azimuth, c is the speed of light, and λ is the wavelength of the signal.

From Equations (1) and (2), the difference between the sub-aperture expression of the signal and the traditional full-aperture expression is only a sub-aperture center term, $t_{k}$, which plays a very important role in image stitching, as detailed in Section 3.2.3.

## 3. Real-Time Imaging Algorithm Based on Sub-Aperture CS-Dechirp

The real-time imaging algorithm based on sub-aperture CS-dechirp can perform imaging while recording data. The data from each sub-aperture are processed by the CS-dechirp algorithm to obtain a low-resolution complex-value image; subsequently, a high-resolution complex-value image of all received data can be obtained by combining all the sub-aperture images coherently.

### 3.1. Description of Real-Time Imaging Algorithm

**Figure 2:**
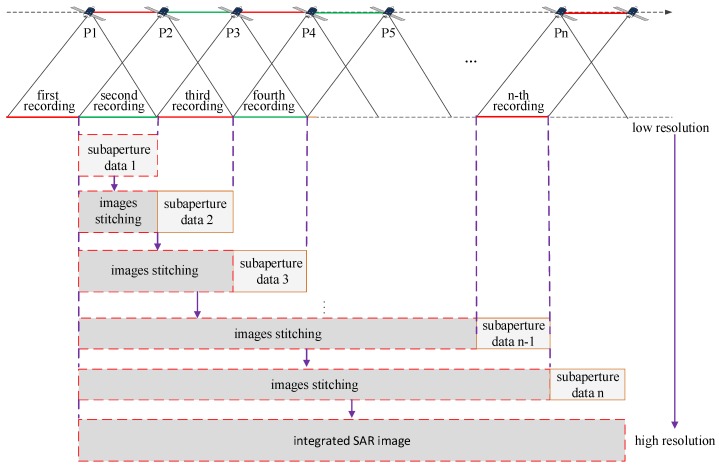
Sketch of the sub-aperture real-time imaging algorithm.

A sketch of the real-time imaging algorithm is shown in Figure 2. The GF3 satellite flies along the trajectory from P1 to Pn, experiencing n times of data recording. During data recording, the corresponding sub-aperture data are processed to form a low-resolution complex-value image, which is then coherently combined with the foregone images to form a new image with higher resolution. As the sub-aperture images are coherently combined one by one, the resolution is gradually improved until full resolution is achieved. This forms the pipeline structure of the sub-aperture data stream, in which the final imaging result can be gradually generated as sub-aperture data are continuously recorded, which is suitable for the GF3-SAR real-time imaging processing.

**Figure 3:**
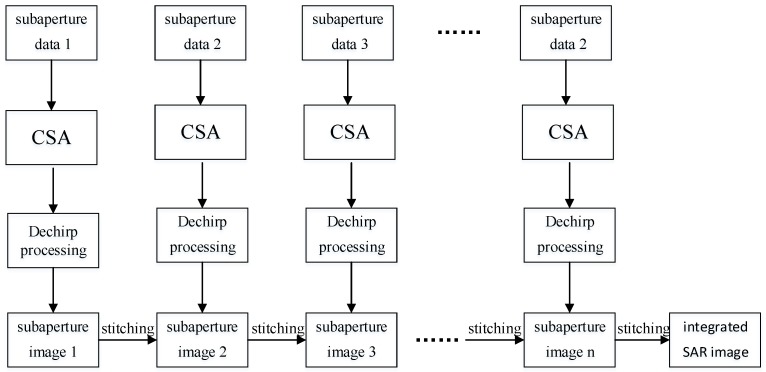
Flow chart of the real-time imaging algorithm.

The flow chart of the real-time imaging algorithm based on sub-aperture CS-dechirp is shown in Figure 3. Data from each sub-aperture recorded by the GF3-SAR are processed by the CS-dechirp algorithm to obtain a low-resolution complex-value image; the high-resolution complex-value image of all received data can then be obtained by stitching together all the sub-aperture images.

### 3.2. Data Processing Based on CS-Dechirp Algorithm

From Equation (1), the slant range $R(t_{sub};R_{B}^{})$ changes with the slow time $t_{sub}$, and the migration amount varies with $R_{B}^{}$, which shows the space-variance characteristic of the range cell migration. Because the scaling processing in the CSA can adequately compensate the range cell migration with the space-variance characteristic and the CSA is suitable for hardware implementation [18], the real-time imaging algorithm takes the CS-dechirp algorithm as the core to process sub-aperture data.

According to the flow chart shown in Figure 3, the data from each sub-aperture go through the sub-aperture CSA module, azimuth dechirp module, and sub-aperture complex-value images stitching module to obtain a SAR image with higher resolution.

#### 3.2.1. Sub-Aperture CSA

Supposing the sub-aperture Doppler frequency corresponding to $t_{sub}$ is $f_{sub}$, the expression of the k-th sub-aperture echo signal shown in Equation (2) can be written in the t–$f_{sub}$ domain as:

(3) $$\begin{array}{cc} s_{k}(t,f_{sub};R_{B}^{}) & =a_{r}(t-\frac{2R(f_{sub};R_{B}^{})}{c})a_{a}(\frac{R_{B}^{}\lambda f_{sub}}{2v^{2}\sqrt{1-{\left(f_{sub}/f_{aM}\right)}^{2}}})\\ & \times \exp[-j\frac{2\pi }{v}R_{B}^{}\sqrt{f_{aM}{}^{2}-f_{sub}{}^{2}}]\exp[-j2\pi f_{sub}(\frac{X}{v}-t_{k})]\\ & \times \exp[j\pi \gamma_{e}(f_{sub};R_{B}^{})(t-\frac{2R(f_{sub};R_{B}^{})}{c}){}^{2}] \end{array}$$

where $\frac{1}{\gamma_{e}(f_{sub};R_{B}^{})}=\frac{1}{\gamma }-R_{B}^{}\frac{2\lambda \sin^{2}\theta }{c^{2}\cos^{3}\theta }$, θ is the squint angle and $f_{aM}=2v/\lambda$. From Equation (3), the difference between the sub-aperture expression of the signal and the traditional full-aperture expression is the second exponent term of $t_{k}$, which shows the phase of the point target relative to $t_{k}$ in sub-aperture imaging. 

The chirp scaling quadratic phase function for the range cell migration correction is:

(4) $$H_{1}(t,f_{sub};R_{0})=\exp[j\pi \gamma_{e}(f_{sub};R_{B}^{})a(f_{sub}){\left(t-\frac{2R(f_{sub};R_{0})}{c}\right)}^{2}]$$

where $a(f_{sub})=1/\sqrt{1-{\left(f_{sub}/f_{aM}\right)}^{2}}-1$ is the CS factor, $R(f_{sub};R_{0})$ is the slant range with $f_{sub}$ as an argument, and $R_{0}$ is the reference distance between the scene center and the radar trajectory. Since $\gamma_{e}(f_{sub};R_{B}^{})$ changes less within the observed scene, $R_{B}^{}$ in $\gamma_{e}(f_{sub};R_{B}^{})$ can be replaced by $R_{0}$. In the following part, we use $\gamma_{e}(f_{sub};R_{0})$ to substitute $\gamma_{e}(f_{sub};R_{B}^{})$.

After multiplying Equations (4) with (3) in the t–$f_{sub}$ domain, the signal is transformed to the $f_{r}$–$f_{sub}$ domain as:

(5) $$\begin{array}{cc} s_{k}(f_{r},f_{sub};R_{B}^{}) & =a_{r}[-\frac{f_{r}}{\gamma_{e}(f_{sub};R_{0})[1+a(f_{sub})]}]a_{a}(\frac{R_{B}^{}\lambda f_{sub}}{2v^{2}\sqrt{1-{\left(f_{sub}/f_{aM}\right)}^{2}}})\\ & \times \exp[-j\pi \frac{f_{r}^{2}}{\gamma_{e}(f_{sub};R_{0})[1+a(f_{sub})]}]\exp[-j\frac{4\pi }{c}[R_{B}^{}+R_{0}a(f_{sub})]f_{r}]\\ & \times \exp[-j\frac{2\pi }{v}R_{B}^{}\sqrt{f_{aM}{}^{2}-f_{sub}{}^{2}}]\exp[-j2\pi f_{sub}(\frac{X}{v}-t_{k})] \end{array}$$

where $\Theta_{\Delta }(f_{sub};R_{B})=\frac{4\pi }{c^{2}}\gamma_{e}(f_{sub};R_{0})a(f_{sub})[1+a(f_{sub})]{\left(R_{B}-R_{0}\right)}^{2}$ is the residual phase due to the operation of the chirp scaling quadratic phase function. The first exponent term in Equation (5) is the modulation phase function in the range frequency domain. $R_{0}a(f_{sub})$ in the second exponent term shows that the migration amount of all point targets in the observed scene becomes the same after the CS operation.

Subsequently, the phase function for the range compressing, secondary range compressing, and range cell migration correction can be constructed as:

(6) $$H_{21}(f_{r},f_{sub};R_{0})=\exp[j\pi \frac{f_{r}^{2}}{\gamma_{e}(f_{sub};R_{0})[1+a(f_{sub})]}]\exp[j\frac{4\pi R_{0}a(f_{sub})}{c}f_{r}]$$

After multiplying Equations (6) with (5), the signal is transformed to the t–$f_{sub}$ domain as follows:

(7) $$\begin{array}{cc} s_{k}(t,f_{sub};R_{B}^{}) & ={\sin c}_{a_{r}}(t-\frac{2R_{B}^{}}{c})a_{a}(\frac{R_{B}^{}\lambda f_{sub}}{2v^{2}\sqrt{1-{\left(f_{sub}/f_{aM}\right)}^{2}}})\\ & \times \exp[-j\frac{2\pi }{v}R_{B}^{}\sqrt{f_{aM}{}^{2}-f_{sub}{}^{2}}]\exp[j\Theta_{\Delta }(f_{sub};R_{B}^{})]\\ & \times \exp[-j2\pi f_{sub}(\frac{X}{v}-t_{k})] \end{array}$$

Here, the CSA completes the range compressing and range cell migration correction. From Equation (7), the first exponent term is required to process matched filtering, and the second exponent term is the residual phase term that is required to compensate. Based on the above, the residual phase compensation function for Equation (7) can be written as:

(8) $$H_{22}(t,f_{sub};R_{0})=\exp[-j\Theta_{\Delta }(f_{sub};R_{B}^{})]$$

**Figure 4:**
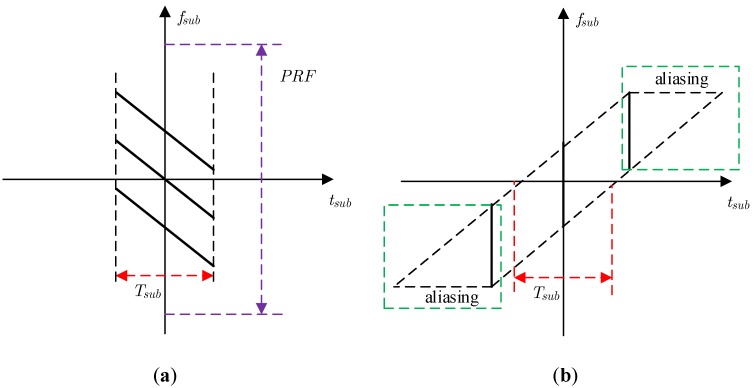
Time-frequency distribution for matched filtering: (**a**) unprocessed sub-aperture signal; (**b**) sub-aperture signal after matched filtering.

**Figure 5:**
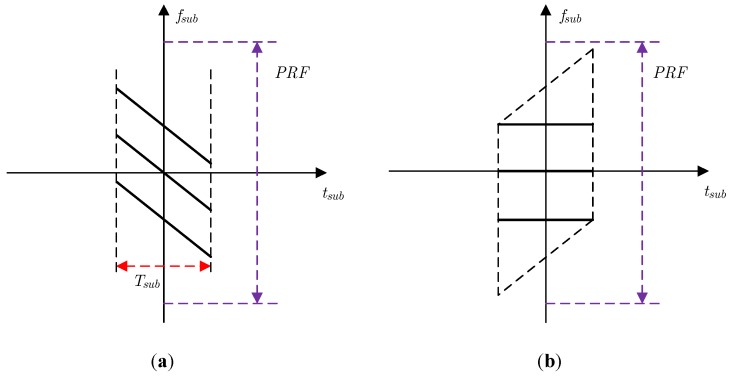
Time-frequency distribution for azimuth dechirp: (**a**) unprocessed sub-aperture signal; (**b**) sub-aperture signal after azimuth dechirp.

However, the matched filtering used for the sub-aperture signal of Equation (7) will cause image aliasing, which can be illustrated by the time-frequency distribution in Figure 4. To avoid this aliasing caused by the matched filtering, zero padding in the azimuth must be used for the traditional CSA, which undoubtedly increases the computation burden. Therefore, the azimuth dechirp can be used for azimuth compression. The azimuth dechirp can solve the problem of image aliasing without zero padding, which can be illustrated by the time-frequency distribution in Figure 5.

#### 3.2.2. Azimuth Dechirp

Since the high-order term in Equation (7) leads to the residual phase in the azimuth dechirp, the high-order term must be converted to a quadratic phase function. The conversion function can be written as:

(9) $$H_{3}(t,f_{sub};R_{B}^{})=\exp[j\frac{2\pi }{v}R_{B}^{}\sqrt{f_{aM}{}^{2}-f_{sub}{}^{2}}-j\frac{\pi }{k_{a0}}f_{sub}{}^{2}]$$

where $k_{a0}=-2v^{2}/\lambda R_{0}$ is the time-domain chirp rate of the signal.

After the compensation of Equation (8) and the conversion of Equation (9), the signal can be written as:

(10) $$\begin{array}{cc} s_{k}(t,f_{sub};R_{B}^{}) & ={\sin c}_{a_{r}}(t-\frac{2R_{B}^{}}{c})a_{a}(\frac{R_{B}^{}\lambda f_{sub}}{2v^{2}\sqrt{1-{\left(f_{sub}/f_{aM}\right)}^{2}}})\\ & \times \exp[-j\frac{\pi }{k_{a0}}f_{sub}{}^{2}-j2\pi (\frac{X}{v}-t_{k})f_{sub}] \end{array}$$

The signal of Equation (10) is transformed to the t–$t_{sub}$ domain as:

(11) $$s_{k}(t,t_{sub};R_{B}^{})={\sin c}_{a_{r}}(t-\frac{2R_{B}^{}}{c})a_{a}(t_{k}+t_{sub}-\frac{X}{v})\exp[j\pi k_{a0}{\left(t_{k}+t_{sub}-\frac{X}{v}\right)}^{2}]$$

From Equation (11), there are only the first and quadratic terms in its phase. A quadratic phase function can be constructed to complete the azimuth dechirp as:

(12) $$H_{4}(t,t_{sub})=\exp(-j\pi k_{a0}\left(t_{k}+t_{sub}\right){}^{2})$$

After the dechirp processing in Equation (12), the signal of Equation (11) becomes:

(13) $$\begin{array}{cc} s_{k}(t,t_{sub};R_{B}^{}) & ={\sin c}_{a_{r}}(t-\frac{2R_{B}^{}}{c})a_{a}(t_{k}+t_{sub}-\frac{X}{v})\\ & \times \exp[-j2\pi k_{a0}\frac{X}{v}(t_{k}+t_{sub})+j\pi k_{a0}(\frac{X}{v}){}^{2}] \end{array}$$

#### 3.2.3. Sub-Aperture Complex-Value Image Stitching

The theoretical form of sub-aperture image stitching can be described as follows. For simplicity, Equation (13) can be rewritten as:

(14) $$\begin{array}{cc} s_{k}(t,t_{sub};R_{B}^{}) & ={\sin c}_{a_{r}}(t-\frac{2R_{B}^{}}{c})a_{a}(t_{k}+t_{sub}-\frac{X}{v})\\ & \times \exp[-j2\pi f_{d}t_{sub}-j2\pi f_{d}t_{k}+j\pi k_{a0}(\frac{X}{v}){}^{2}] \end{array}$$

where $f_{d}=k_{a0}X/v$.

According to Equation (14), the Fourier transformation (FT) must be performed on the azimuth signal to obtain the sub-aperture focusing results. However, the focusing position $f_{d}$ in the azimuth exceeds the range of (−PRF/2, PRF/2) (PRF, Pulse Repeating Frequency), resulting in azimuth-aliasing after the FT for the signal. Therefore, the FT cannot be directly performed. To deal with this problem, an azimuth compensation function for spectrum shifting is introduced:

(15) $$H_{5}(t,t_{sub})=\exp(j2\pi f_{M}t_{sub})$$

where $f_{M}=k_{a0}t_{k}$. This function ensures that the focusing positions of point targets are within (−PRF/2, PRF/2). Multiplying Equations (15) with (14), the signal becomes:

(16) $$\begin{array}{cc} s_{k}(t,t_{sub};R_{B}^{}) & ={\sin c}_{a_{r}}(t-\frac{2R_{B}^{}}{c})a_{a}(t_{k}+t_{sub}-\frac{X}{v})\\ & \times \exp[-j2\pi (f_{d}-f_{M})t_{sub}-j2\pi f_{d}t_{k}+j\pi k_{a0}(\frac{X}{v}){}^{2}] \end{array}$$

Then, the signal is transformed to the t–$f_{sub}$ domain as:

(17) $$\begin{array}{cc} s_{k}(t,f_{sub};R_{B}^{}) & ={\sin c}_{a_{r}}(t-\frac{2R_{B}^{}}{c})\delta_{a}(f_{sub}+f_{d}-f_{M})\\ & \times \exp(-j2\pi f_{d}t_{k})\exp(j\pi k_{a0}(\frac{X}{v}){}^{2}) \end{array}$$

Because the focus position $f_{M}-f_{d}$ is smaller than PRF, the single in Equation (17) is non-azimuth-aliasing. 

In addition, a coherent combination is required for sub-aperture images. Therefore, the phase coherence and simplicity of the combination also need to be considered when performing the above frequency compensation. Thus, a combination method based on the integer points of the azimuth frequency is proposed.

According to Equation (17), one can note that $\exp(-j2\pi f_{d}t_{k})$ is a residual phase, which varies with $t_{k}$ or the azimuth sub-aperture index k. To combine the sub-aperture complex-value images coherently, this phase term should be compensated, and the phase compensation function can be written as:

(18) $$H_{6}(t,f_{sub})=\exp(j2\pi (f_{M}-f_{sub})t_{k})$$

After the compensation of Equation (18), the SAR complex-value image of the k-th sub-aperture data can be obtained as:

(19) $$s_{k}(t,f_{sub};R_{B}^{})={\sin c}_{a_{r}}(t-\frac{2R_{B}^{}}{c})\delta_{a}(f_{sub}+f_{d}-f_{M})\exp(j\pi k_{a0}{\left(\frac{X}{v}\right)}^{2})$$

## 4. Simulation and Real Data Results

In order to illustrate the effectiveness of the proposed algorithm, the imaging results of a point targets simulation and the GF3-SAR real data, which are separately processed by the full-aperture standard CSA and the sub-aperture CS-dechirp algorithm, are used for comparative analysis.

### 4.1. Point Targets Simulation

The simulation parameters of the spaceborne SAR are shown in Table 1 and the lattice in the scene is 3 (range) × 5 (azimuth). The distance between point targets is 1500 m in the range and 625 m in the azimuth.

**Figure 6:**
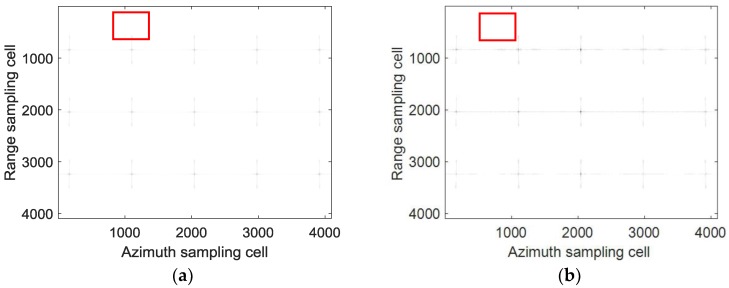
Imaging results of point targets: (**a**) standard full-aperture chirp scaling algorithm (CSA); (**b**) sub-aperture chirp scaling (CS)-dechirp algorithm.

**Figure 7:**
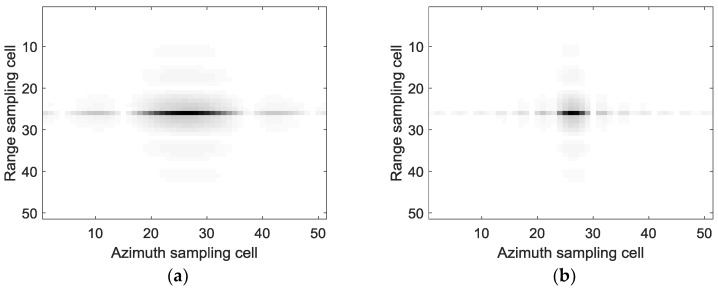

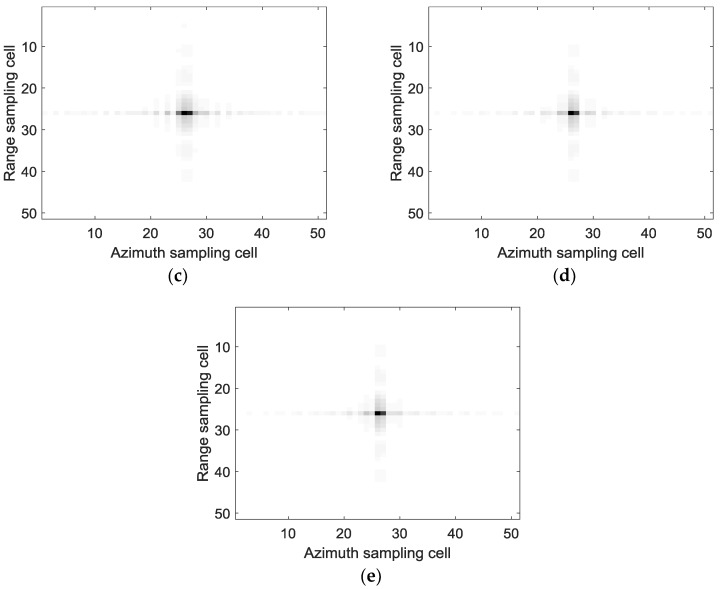
Imaging results after sub-aperture synthesis. (**a**) With one sub-aperture datum; (**b**) With two sub-aperture data; (**c**) With three sub-aperture data; (**d**) With four sub-aperture data; (**e**) With five sub-aperture data.

**Figure 8:**
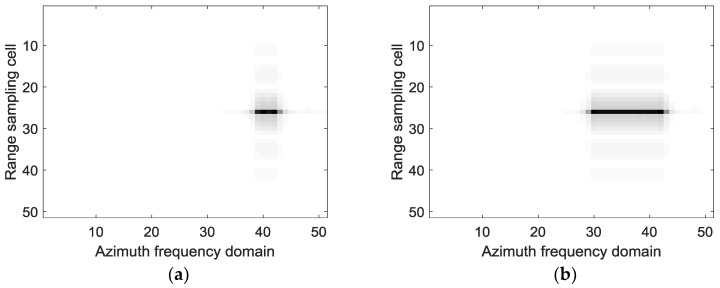

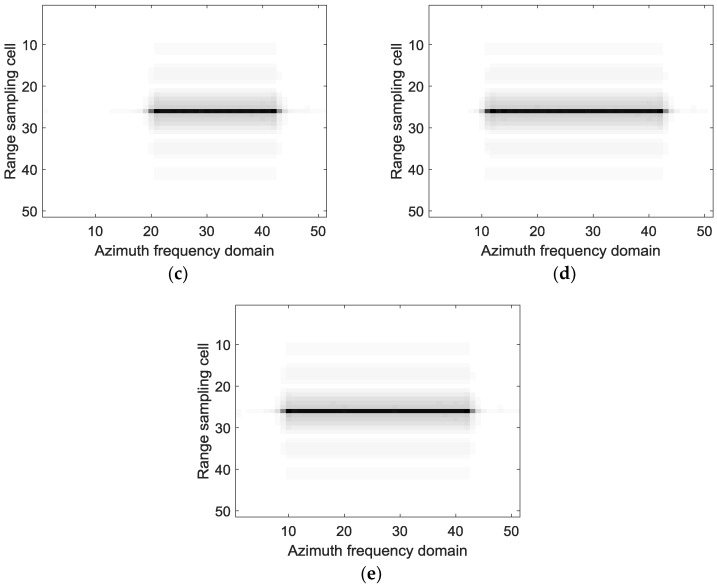
Frequency spectrum of the azimuth signal after sub-aperture synthesis. (**a**) With one sub-aperture datum; (**b**) With two sub-aperture data; (**c**) With three sub-aperture data; (**d**) With four sub-aperture data; (**e**) With five sub-aperture data.

The imaging results with the standard full-aperture CSA and the sub-aperture CS-dechirp algorithm are shown in Figure 6. To show the process of sub-aperture data imaging and coherent sub-aperture stitching, the focusing results and corresponding azimuth spectrums of the point target in the red rectangle of Figure 6b are shown in Figure 7 and Figure 8. Figure 7 and Figure 8 show the change in focusing results and azimuth spectrums as the sub-aperture images are combined. In this simulation, the length of the sub-aperture is one-fifth of the full-aperture length. The focusing results of 1–5 sub-apertures are presented in Figure 7a–e. From Figure 7, one can note that the resolution of the point target is gradually improved as the sub-aperture synthesis amount increases.

Figure 8a–e, corresponding to Figure 7a–e, show the change in the azimuth frequency spectrum with sub-aperture combination. From Figure 8a–e, the bandwidths of the azimuth frequency spectrum are gradually increased as the sub-aperture synthesis amount increases. From Figure 7 and Figure 8, it can be seen that the sub-aperture image combination in our method is equivalent to the coherent combination of the azimuth spectrum.

**Figure 9:**
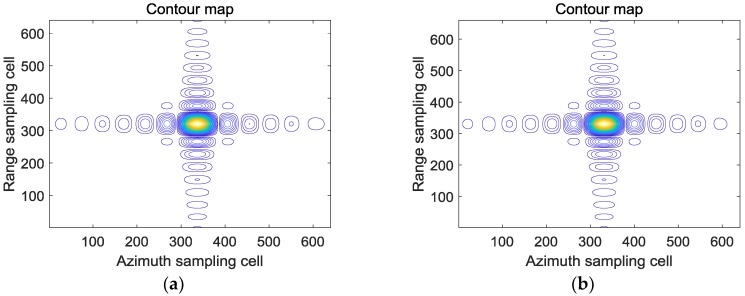

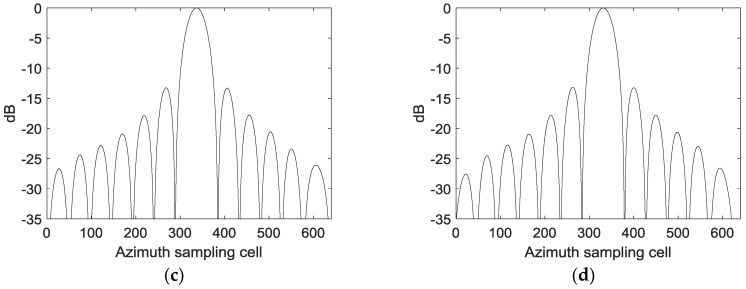
Results of the standard full-aperture CSA and the proposed method: (**a**) standard CSA; (**b**) proposed method; (**c**) azimuth profiles based on the standard CSA; (**d**) azimuth profiles based on the proposed method.

**Figure 10:**
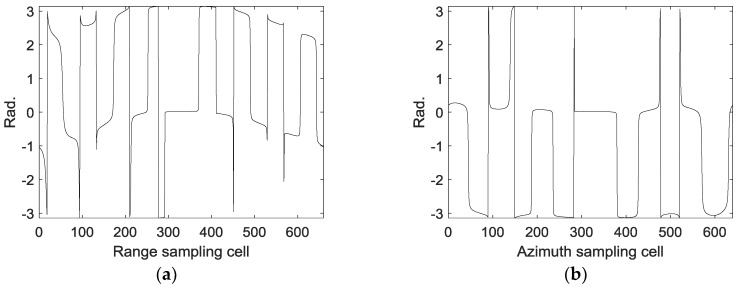
Phase of the point target processed by the proposed algorithm: (**a**) phase in range; (**b**) phase in azimuth.

The contours and azimuth profiles of the point targets marked by the red box in Figure 6a,b is show in Figure 9. From Figure 9, the focusing result processed by the sub-aperture CS-dechirp algorithm is consistent with the standard CSA, which proves that the proposed real-time imaging algorithm in this paper is feasible. Figure 10 shows the phase of the point target processed by the sub-aperture CS-dechirp algorithm in the range and azimuth, indicating the good coherence of the sub-aperture signal. Since the high-order phase is compensated by the sub-aperture CS-dechirp method and the sub-aperture overlapping is avoided, the grating lobes do not appear in our method. In other words, our method has better performance in grating lobe suppression than that in Reference [16].

### 4.2. GF3-SAR Data Results

**Figure 11:**
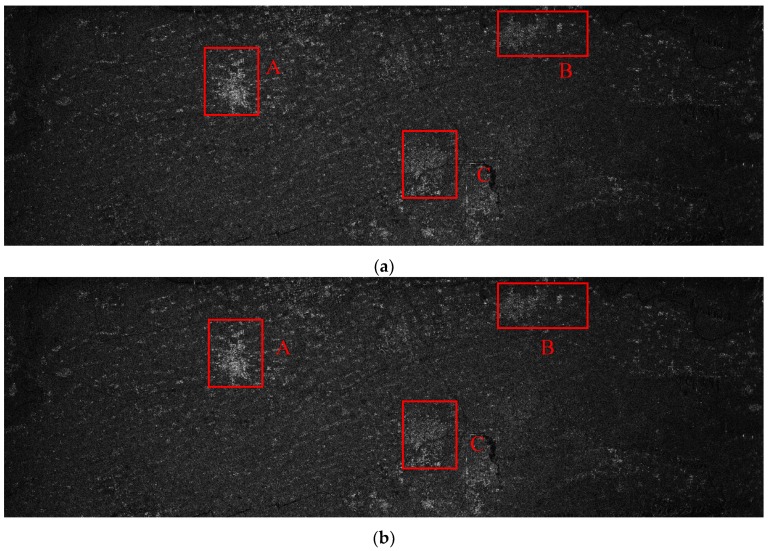
Imaging results of the GF3-SAR stripmap data (from **top** to **bottom** is range, and from **left** to **right** is azimuth): (**a**) standard full-aperture CSA; (**b**) proposed algorithm.

**Figure 12:**
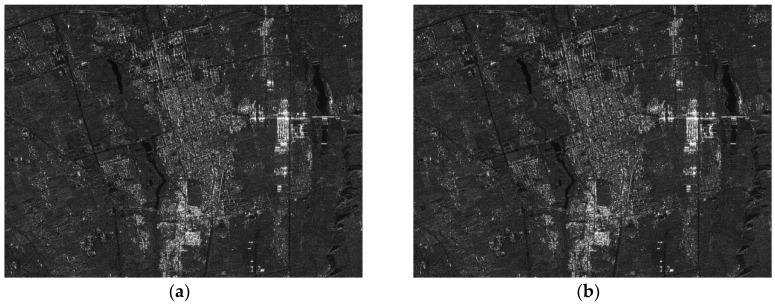
Results of area A (from **top** to **bottom** is range, and from **left** to **right** is azimuth): (**a**) standard full-aperture CSA; (**b**) proposed algorithm.

**Figure 13:**
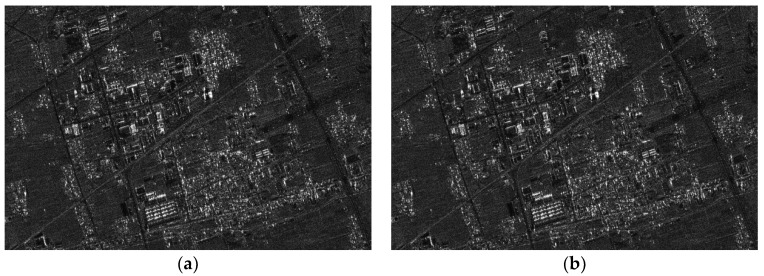
Results of area B (from **top** to **bottom** is range, and from **left** to **right** is azimuth): (**a**) standard full-aperture CSA; (**b**) proposed algorithm.

**Figure 14:**
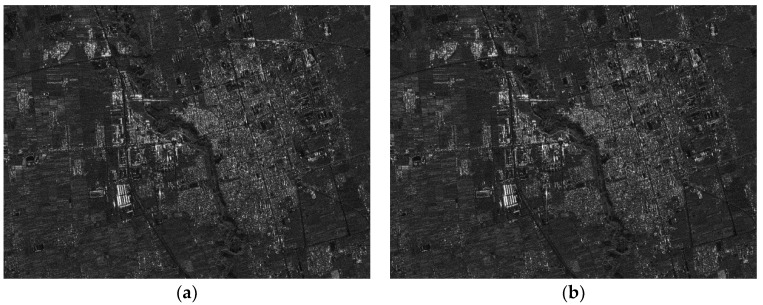
Results of area C (from **top** to **bottom** is range, and from **left** to **right** is azimuth): (**a**) standard full-aperture CSA; (**b**) proposed algorithm.

The parameters of the GF3-SAR with stripmap mode are shown in Table 2. The imaging result based on the standard full-aperture CSA is shown in Figure 11a and that based on the sub-aperture CS-dechirp algorithm is shown in Figure 11b. By enlarging the area marked by the red box in Figure 11a,b, detailed images are shown in Figure 12, Figure 13 and Figure 14. From the detailed images, one can note that the result processed by the sub-aperture CS-dechirp algorithm is consistent with the standard full-aperture CSA.

### 4.3. Computational Load Analysis

Assuming the final image is of $N_{r}\times N_{a}$ pixels, where $N_{r}$ and $N_{a}$ represent the range and azimuth points, respectively, the computational complexities of the standard full-aperture CSA and the proposed method are analyzed as follows. The floating-point operations can be derived as $5N_{r}N_{a}\log_{2}^{}N_{r}$ for FFT in range and $5N_{r}N_{a}\log_{2}^{}N_{r}$ for FFT in azimuth. The complex multiplication needs $6N_{r}N_{a}$ operations [19].

For the standard full-aperture CSA, the processing system processes all recorded data. The standard full-aperture CSA contains four FFTs and three complex multiplications. Then, its computational complexity can be achieved by:

(20) $$S_{csa}=10N_{r}N_{a}\log_{2}^{}N_{r}+10N_{r}N_{a}\log_{2}^{}N_{a}+18N_{r}N_{a}$$

For the proposed method, the processing system processes data from one sub-aperture each time. The proposed method contains five FFTs and five complex multiplications. Assuming the sub-aperture number in the azimuth is N, the number of azimuth points of sub-aperture data is $N_{a}/N$. Then, the computational complexity for one sub-aperture data can be achieved by:

(21) $$\begin{array}{cc} S_{sub} & =10N_{r}\frac{N_{a}}{N}\log_{2}^{}N_{r}+15N_{r}\frac{N_{a}}{N}\log_{2}^{}N_{a}/N+30N_{r}\frac{N_{a}}{N}\\ & =\frac{10}{N}N_{r}N_{a}\log_{2}^{}N_{r}+\frac{15}{N}N_{r}N_{a}\log_{2}^{}N_{a}+\frac{30}{N}N_{r}N_{a}-\frac{15}{N}N_{r}N_{a}\log_{2}^{}N \end{array}$$

Thus, the computational complexity of the proposed method is:

(22) $$\begin{array}{cc} S_{pro} & =N\cdot S_{sub}\\ & =10N_{r}N_{a}\log_{2}^{}N_{r}+15N_{r}N_{a}\log_{2}^{}N_{a}+30N_{r}N_{a}-15N_{r}N_{a}\log_{2}^{}N \end{array}$$

The magnitudes of $S_{csa}$ and $S_{pro}$ are similar, so the computational load of the proposed method is almost the same as that of the standard full-aperture CSA. However, the proposed method can perform imaging processing while recording data; therefore, the proposed method based on the pipeline structure has a relatively high computational efficiency.

## 5. Conclusions

The GF3-SAR real-time imaging algorithm based on sub-aperture CS-dechirp can perform imaging processing while recording data, which reduces the amount of data storage and computational load for the GF3-SAR system and has good real-time performance. In order to illustrate the effectiveness of the algorithm proposed in this paper, the imaging results of point targets simulation and GF3-SAR real data, which are separately processed, are used for comparative analysis. The algorithm based on sub-aperture CS-dechirp is very suitable for the real-time imaging processing of the GF3-SAR.

## Figures and Tables

**Table 1:** Simulation parameters of the spaceborne SAR.

Parameter	Value
Carrier frequency	9.63 GHz
Bandwidth	50 MHz
Sample frequency	60 MHz
Velocity	7391 m/s
PRF	2738 Hz
Center line distance	617 km

**Table 2:** Parameters of the GF3-SAR in stripmap mode.

Parameter	Value
Bandwidth	100 MHz
Sample frequency	133 MHz
Wavelength	0.055 m
Velocity	7132 m/s
PRF	2580 Hz
Center line distance	842 km
